# A Novel Receptor Tyrosine Kinase Switch Promotes Gastrointestinal Stromal Tumor Drug Resistance

**DOI:** 10.3390/molecules22122152

**Published:** 2017-12-05

**Authors:** Sergei Boichuk, Aigul Galembikova, Pavel Dunaev, Elena Valeeva, Elena Shagimardanova, Oleg Gusev, Svetlana Khaiboullina

**Affiliations:** 1 Department of Pathology, Kazan State Medical University, Kazan 420012, Russia; ailuk000@mail.ru (A.G.); dunaevpavel@mail.ru (P.D.); vevaleeva@ya.ru (E.V.); 2 Kazan Federal (Volga Region) State University, Kazan 420008, Russia; rjuka@mail.ru (E.S.); gaijin@gmail.com (O.G.); sv.khaiboullina@gmail.com (S.K.); 3 Innovation Center, RIKEN, Yokohama 230-0045, Japan; 4 Preventive Medicine and Diagnosis Innovation Program, RIKEN, Yokohama 230-0045, Japan

**Keywords:** gastrointestinal stromal tumor cells (GISTs), imatinib (IM), FGFR2α, resistance, receptor tyrosine kinase (RTK) inhibitors, chemotherapeutic drugs

## Abstract

The fact that most gastrointestinal stromal tumors (GISTs) acquire resistance to imatinib (IM)-based targeted therapy remains the main driving force to identify novel molecular targets that are capable to increase GISTs sensitivity to the current therapeutic regimens. Secondary resistance to IM in GISTs typically occurs due to several mechanisms that include hemi- or homo-zygous deletion of the wild-type KIT allele, overexpression of focal adhesion kinase (FAK) and insulin-like growth factor receptor I (IGF-1R) amplification, BRAF mutation, a RTK switch (loss of c-KIT and gain of c-MET/AXL), etc. We established and characterized the IM-resistant GIST T-1 cell line (GIST T-1R) lacking secondary c-KIT mutations typical for the IM-resistant phenotype. The resistance to IM in GIST T-1R cells was due to RTK switch (loss of c-KIT/gain of FGFR2α). Indeed, we have found that FGFR inhibition reduced cellular viability, induced apoptosis and affected the growth kinetics of the IM-resistant GISTs in vitro. In contrast, IM-naive GIST T-1 parental cells were not susceptible to FGFR inhibition. Importantly, inhibition of FGF-signaling restored the susceptibility to IM in IM-resistant GISTs. Additionally, IM-resistant GISTs were less susceptible to certain chemotherapeutic agents as compared to parental IM-sensitive GIST cells. The chemoresistance in GIST T-1R cells is not due to overexpression of ABC-related transporter proteins and might be the result of upregulation of DNA damage signaling and repair (DDR) genes involved in DNA double-strand break (DSB) repair pathways (e.g., XRCC3, Rad51, etc.). Taken together, the established GIST T-1R cell subline might be used for in vitro and in vivo studies to examine the efficacy and prospective use of FGFR inhibitors for patients with IM-resistant, un-resectable and metastatic forms of GISTs with the type of RTK switch indicated above.

## 1. Introduction

Gastrointestinal stromal tumors (GISTs) are thought to originate from the specialized cells in the bowel wall, the interstitial cells of Cajal (ICCs), or their stem cell-like precursors. These cells are the “pacemakers” of the gastrointestinal tract that ensure the peristalsis of the intestinal wall. Since most of GISTs are caused by activating mutations of the KIT receptor tyrosine kinase gene in ICCs [1], the tyrosine kinase inhibitor imatinib mesylate (IM) is considered as the most effective first-line treatment option for patients with advanced, unresectable and/or metastatic forms of GISTs [2,3]. However, despite the impressive response rates, more than half of the patients with GIST develop IM resistance during the first two years of treatment [4,5]. Secondary resistance to IM typically occurs due to several known mechanisms. These include hemi- or homo-zygous deletion of the wild-type KIT allele [6], overexpression of focal adhesion kinase (FAK) [7] and insulin-like growth factor receptor I (IGF-1R) amplification [8], BRAF V600E mutation (5% GIST) [9] and RTK switch (loss of c-KIT and gain of MET/AXL) [10], etc. Recently, it was shown that FGFR-signaling in GISTs significantly attenuated anti-tumor effects of IM, thus providing the evidence about the cross-talk between KIT and FGFR playing an important role in IM resistance [11,12]. To overcome the resistance to IM, more potent KIT inhibitors were developed. However, the second- and third-generation of multi-kinase inhibitors, sunitinib malate (Sutent) [13] and regorafenib (Stivarga) [14], offered limited additional benefits for GISTs patients, thus indicating the need to develop effective therapeutic options to prevent disease progression, delay the resistance to therapy and achieve symptomatic benefits.

Given that RTK switch is one of the mechanisms underlying IM resistance in GISTs, we established the IM-resistant GIST T-1 cell subline (GIST T-1R) where FGFR2α and MET RTKs were overexpressed. Additionally, the GIST T1-R cells lack c-KIT secondary mutations responsible for secondary IM-resistance. Next, we examined the sensitivity of these cells to the corresponding RTK inhibitors and to the DNA damaging agents, topoisomerase type II inhibitors, microtubule-affecting drugs, etc.

Our data indicate that selective FGFR inhibitor BGJ398 is effective against IM-resistant GIST T-1R cells with the RTK switch (c-KIT/FGFR2α) Of note, crizotinib (CR) and cabozantinib (CB) as potent c-MET-inhibitors also reduced the viability of IM-resistant GISTs, which is consistent with c-MET overexpression observed in this GIST subline. However, since phospho-FGFR2α expression was substantially decreased in CB-treated GIST T-1R cells, the inhibitory effect of CB in GIST T-1R cells could be also due to the attenuation of FGF-signaling.

Importantly, the inhibitors indicated above enhanced sensitivity of IM-resistant GISTs to IM. Interestingly, IM’s resistance also affected GISTs sensitivity to the chemotherapeutic drugs, such as doxorubicin, etoposide, paclitaxel, vinblastine, etc. The resistance was not due to the overexpression of the ABC-transporter-related proteins typically responsible for the multi-drug resistance (MDR) phenotype, but rather a consequence of increased DNA damage repair (DDR) capacities in IM-resistant GISTs.

Taken together, our data indicate the prospective use of the multi-kinase inhibitors of c-MET (crizotinib, cabozantinib, etc.) and FGFR (ponatinib, dovitinib, etc.) for GIST patients with secondary resistance to IM due to this type of RTK switch. Importantly, the IM-resistant GISTs with this type of RTK switch might be less sensitive to the conventional chemotherapy when compared to the patients with IM-sensitive tumors.

## 2. Results

### 2.1. Establishment and Characterization of the IM-Resistant GIST T-1R Cell Subline

**Figure 1:**
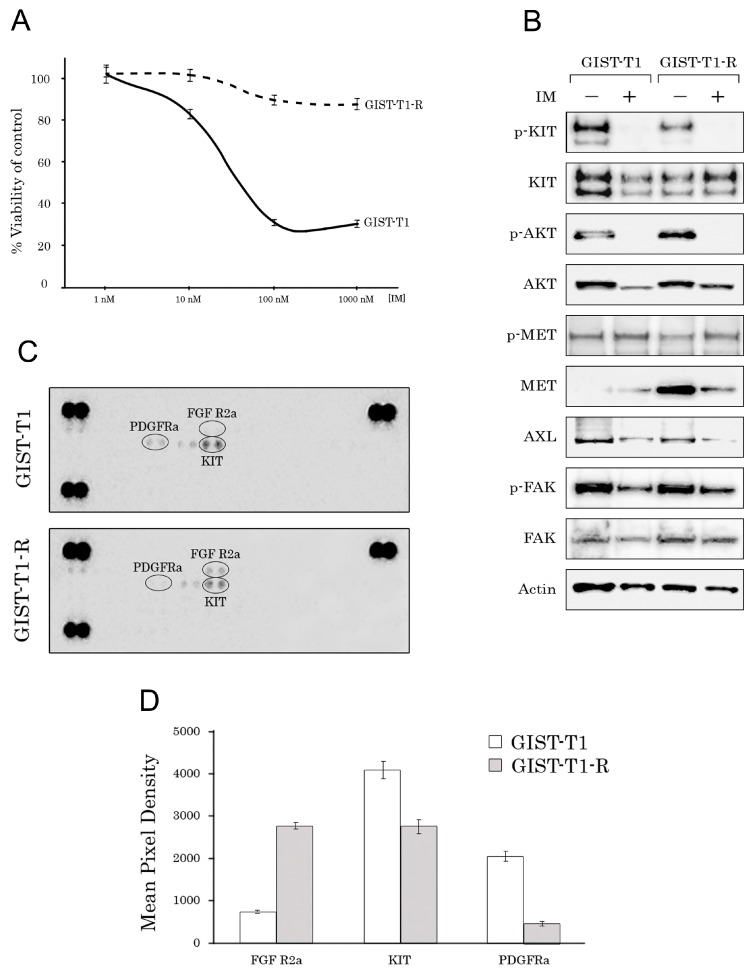
(**A**) MTS-based viability assay in IM-treated GIST-T1 vs. GIST T-1R (R, resistant) cells. Cells were treated with the indicated concentrations of IM and assessed after 48 h of treatment, with the data normalized to DMSO-treated controls. Values are the means ± standard deviation (n = 3); (**B**) Immunoblot evaluations of KIT, AKT, MET, AXL and FAK in non-treated (−) and IM-treated (+) GIST T-1 and GIST T-1R cells. Actin stain is a loading control; (**С**) Total cell lysates (300 µg) from GIST T-1 (upper panel) and GIST T-1R cells (lower panel) were analyzed by the phospho-RTK array. Each RTK was spotted in duplicate, and the spots at each corner are positive controls; (**D**) Quantification by mean pixel density revealed that the phosphorylated proteins are dysregulated in GIST T-1R vs. parental GIST T-1 cells.

The IM-resistant GIST T-1 cell line (GIST-T1R) was established after a continuous induction from 0.4 nM–1000 nM imatinib (IM) in a stepwise increasing concentration manner for 54 weeks. The IC_50_ for IM in the GIST T-1 cell line was 1.19 ± 0.12 μM. In contrast, the GIST T-1R subline was highly resistant to IM, and no IC_50_ values were detected when IM was used at concentrations up to 10 μM (Figure 1A). However, decreased KIT and AKT phosphorylation was observed in GIST T-1R cells after IM treatment (Figure 1B). IM resistance in the GIST T1-R subline remained stable following storage at −80 °C for three months (data not shown).

To identify the molecular mechanisms of IM resistance in the GIST T-1R subline, we initially performed the analysis of KIT gene in GIST T-1 and GIST T-1R cells using next generation sequencing to examine whether secondary KIT mutations became detectable in the IM-resistant GIST subline. The characteristic deletion in the region inside exon 11 was found in both GIST cell lines. However, the well-known KIT mutations inducing IM resistance were not detected in either GIST T-1 or GIST T-1R cells. Additionally, in GIST T-1R cells, we found several known nucleotide polymorphisms (rs999020, rs995030, rs995029, rs378218 and rs1008658) that likely have no effects on cell resistance to IM. All SNPs (except for rs378218) were detected in both (e.g., IM-naive vs. resistant) GIST cell lines. The rs378218 was present in the heterozygotic form exclusively in GIST-T1R cells. No relationship between this polymorphic site and IM resistance was reported so far, suggesting that resistance to IM in GIST T-1R is not connected to the acquisition of secondary mutation in the KIT gene.

Thus, we concluded that there were no secondary KIT mutations affecting IM-resistance in the GIST T-1R subline. After excluding this fact rendering GIST T-1R cells less susceptible to targeted drug IM, we investigated whether aberrant hyperactivation of the other types of RTKs was responsible for IM resistance in GIST T-1R cells. For this purpose, we utilized phospho-proteome assays (Human Phospho-RTK Array Proteome Profiler™, ARY001B), which allows for simultaneous detection of 42 RTKs in a single antibody-based microarray. Representative images of IM-sensitive GIST T-1 parental cells and their IM-refractory GIST T-1R derivate are shown in Figure 1C. Of the 42 RTKs analyzed via phospho-array, we found that FGFR2α was activated (i.e., phosphorylated) in the IM-resistant GIST T-1R subline, whereas the expression of pKIT and pPDGFRA was substantially downregulated in the IM-refractory GIST T-1R subline when compared to IM-sensitive GIST T-1 parental cells (Figure 1D). There was no difference between these GIST cell lines in the expression of the other activated RTKs, including VEGFR1-3, EGFR, AXL, MET, ALK, Tie-2 and RET.

Western blotting analysis revealed that both GIST cells expressed MET, AXL and FAK; however, the IM-resistant GIST cell line had less total and activated KIT. Furthermore, the total MET expression was ~200-fold higher in the IM-resistant GIST T-1 cell line as compared to IM-sensitive GIST cells (Figure 1B). These data are consistent with the previous reports suggesting that the so-named RTK switch (loss of c-KIT/gain of c-MET) could be responsible for IM resistance of GIST T-1R cells lacking secondary KIT mutations [10,15]. We also found IM-induced MET phosphorylation at Tyr1230/1234/1235 in both GIST cell lines (Figure 1B), supporting the previous report indicating IM’s ability to activate c-MET signaling [16]. Expression of the total and activated forms of the other types of RTKs was not affected in GIST cells exposed to IM (Figure 1B).

Taken together, we established the IM-resistant GIST T1-R cell line lacking secondary KIT mutations rendering IM resistance. Given that IM-resistant GIST T-1R cells overexpressed FGFR2α and MET and taking into account that the RTK switch is a one of the mechanism underlying the IM resistance of GISTs, we further examined whether inhibition of FGFR2a and MET might have a prospective use against the IM-resistant GIST subset with this type of RTK switch.

### 2.2. MET Inhibition Enhances the Cytotoxic Effects of IM in GIST T-1R Cells 

**Figure 2:**
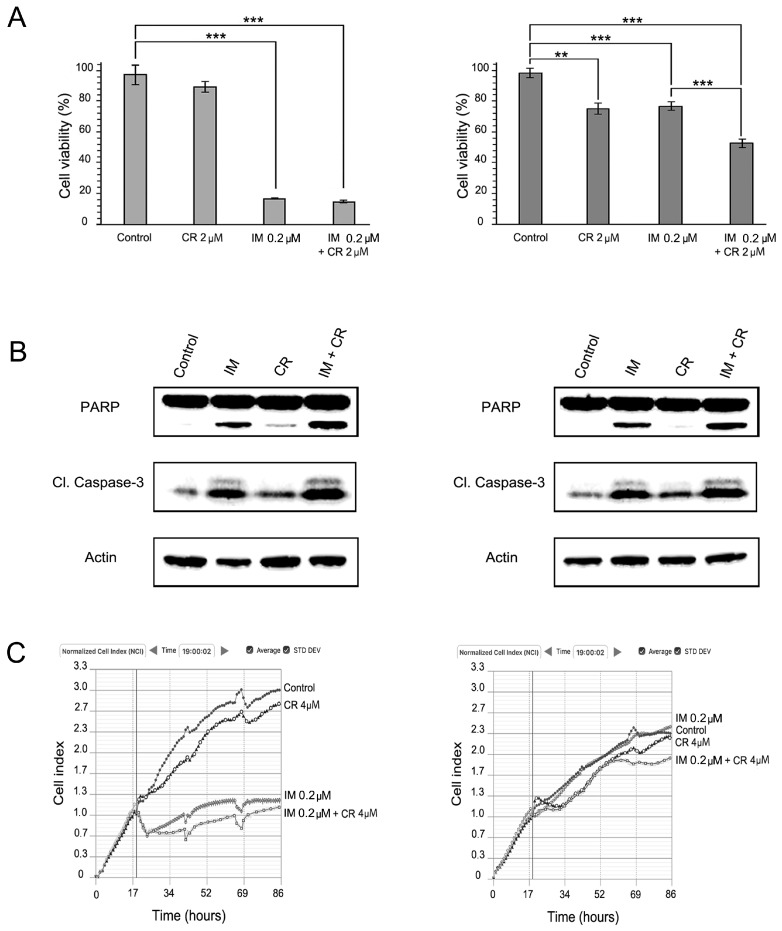
Crizotinib (CR) enhances cytotoxic effects of IM in GIST cells. (**A**) MTS-based viability assay of GIST T-1 and GIST T-1R cells. Treatment with DMSO (control), IM or crizotinib (CR) alone and in combination in GIST T-1 (left) and GIST T-1R (right) cells. Experiments are conducted in triplicates and represented as the mean ± SD. **: *p* ≤ 0.01; ***: *p* ≤ 0.001; (**B**) Immunoblot analysis for apoptosis markers (cleaved forms of PARP and caspase-3) in GIST cells after treatment with DMSO (control), IM, CR alone and in combination (e.g., IM + CR) for 72 h. Actin was used as a loading control; (**C**) Changes in growth kinetics of GIST T-1 (left) and GIST T-1R (right) cells treated with DMSO (control), IM or CR alone and in combination.

To verify the effects of dual KIT and MET inhibition on IM-resistant GISTs, we initially tested crizotinib (CR), a small-molecule inhibitor approved for non-small lung cancer that has selective activity against MET and ALK [17,18]. In GIST T1-R cells, CR induced a moderate decrease of cellular viability when used alone (Figure 2A, right panel). As expected, IM has minor cytotoxic effects on viability of GIST T-1R cells. Importantly, we observed an increased lethality of IM-resistant GIST cells treated with both RTK inhibitors together when compared to GIST T1-R cells treated with CR or IM alone (Figure 2A, right panel). Immunoblot analysis revealed an increased apoptosis in GIST T-1R cells treated with CR in the presence of IM when compared to the cells treated with IM or CR alone (Figure 2B, right panel).

In contrast, CR was non-effective in IM-sensitive GIST T-1 cells when used alone and also did not enhance cytotoxic and pro-apoptotic effects of IM (Figure 2A,B, left panel). The latter might be due to low MET expression in IM-sensitive GIST cells as compared to IM-resistant GIST T1-R derivate (Figure 1B). This could be also due to high level of lethality of GIST T-1 cells after IM treatment. As expected, IM effectively inhibited the growth of GIST T-1 cells and has no inhibitory effects on GIST T-1R cells (Figure 1C). Interestingly, when IM was used in combination with CR, decreased growth kinetics of IM-resistant GISTs was observed (Figure 2C, right panel). Of note, CR has no inhibitory effects on the growth kinetics in IM-sensitive GIST cells when used alone (Figure 2C, left panel).

Next, we tested cabozantinib (CB), a TKI that targets VEGF receptors (VEGFRs), MET, AXL, TIE-2, RET and other RTKs involved in tumor development and progression through angiogenesis, invasiveness, metastasis, anti-apoptosis and drug resistance [19]. Based on the activities indicated above, this multi-RTK inhibitor has been evaluated for the number of solid tumors and recently approved for treatment of medullary thyroid cancer [20] and as a second-line therapy for renal cell carcinoma [21].

**Figure 3:**
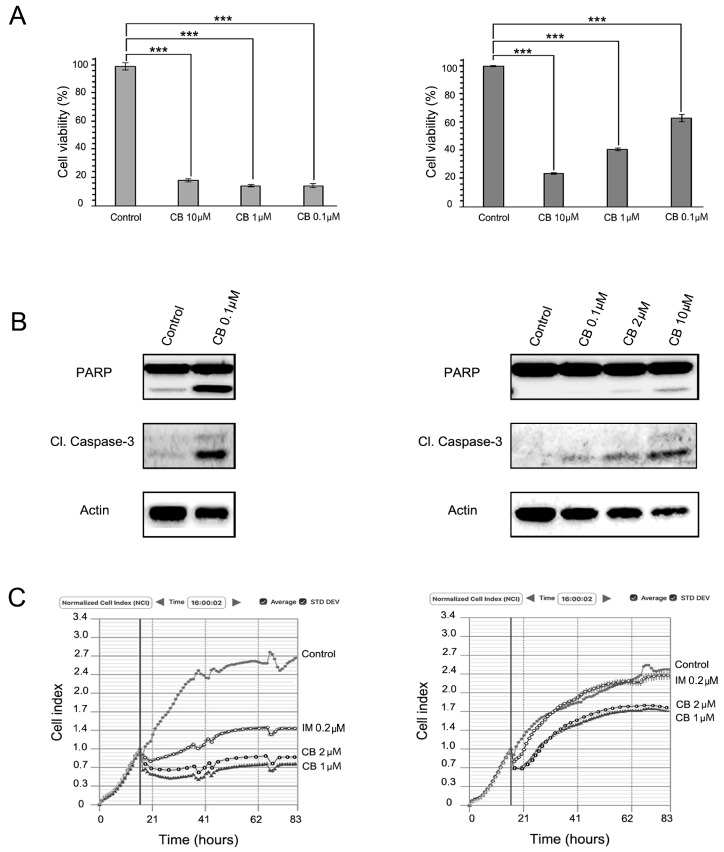
Cabozantinib (CB) inhibits proliferation, growth kinetics and induces apoptosis in IM-sensitive and IM-resistant GIST cells. (**A**) MTS-based viability assay of GIST T-1 and GIST T-1R cells. Treatment with DMSO (control) or CB in GIST T-1 (left) and GIST T-1R (right) cells. Data of triplicates are represented as the mean ± SD. ***: *p* ≤ 0.001; (**B**) Immunoblot analysis for apoptosis markers (cleaved forms of PARP and caspase-3) in GIST cells after treatment with DMSO (control) or CB for 72 h. Actin stain used as a loading control; (**C**) Changes in growth kinetics of GIST T-1 (left) and GIST T-1R (right) cells treated with DMSO (control), IM (positive control) or CB.

We observed that CB substantially decreased cell viability of IM-sensitive GIST T-1 cells (Figure 3A, right panel). Importantly, CB was also effective against IM-resistant GIST T-1R cells: the RTK inhibitor provided a dose-dependent cytotoxic effect (Figure 3A, left panel), induced apoptosis (Figure 3B, left panel) and affected the growth kinetics in GIST T-1R cells (Figure 3C, left panel). However, the effective IC_50_ doses for GIST T-1R cells were much higher, when compared to parental GIST T-1 cells. Similarly, CB concentrations required to induce apoptosis in GIST T-1R cells were ~100-fold higher when compared to GIST T-1 parental cells (Figure 3B).

### 2.3. FGFR Inhibition Substantially Enhances the Cytotoxic Effects of IM in IM-Resistant GISTs

**Figure 4:**
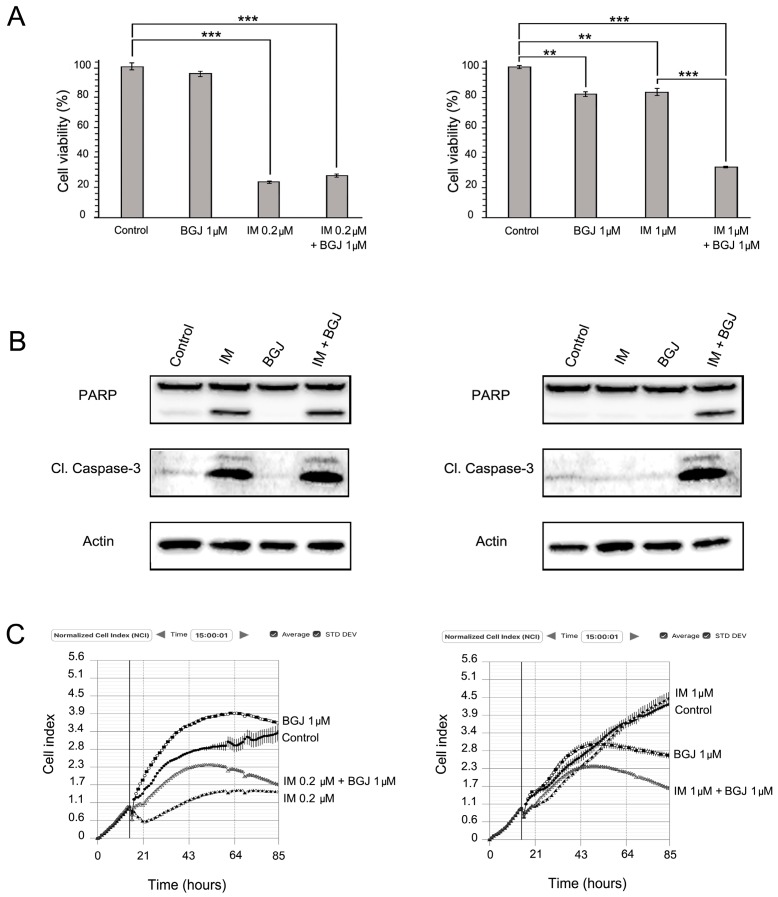
FGFR inhibition induces apoptosis and enhances cytotoxic effects of IM in IM-resistant GIST cells. (**A**) Effects of FGFR inhibition on the viability of GIST T-1 and T-1R cell lines. Cells were treated with DMSO (control), FGFR inhibitor (BGJ398), IM alone and in combination and analyzed using the MTS-based assay after 48 h. Data of triplicates are represented as the mean ± SD. **: *p* ≤ 0.01; ***: *p* ≤ 0.001; (**B**) Immunoblot analysis for apoptosis markers (cleaved forms of PARP and caspase-3) in GIST cells after treatment with DMSO (negative control), BGJ398 or IM alone and in combination for 72 h. Actin stain used as a loading control; (**C**) Changes in growth kinetics of GIST T-1 (left) and GIST T-1R (right) cells treated with DMSO (control), IM or BGJ398 alone and in combination.

Given that phospho-FGFR2α was the major phospho-RTK overexpressed in GIST T-1R cells, we examined whether BGJ398 (selective inhibitor for FGFR1/2/3) is able to reduce viability in IM-sensitive vs. resistant GIST T-1 cells and enhance the cytotoxic effect of IM. We found that FGFR inhibition reduced the viability of IM-resistant GIST T-1R cells, but has no effect on the IM-sensitive GIST T-1 parental cell line (Figure 4A). Despite this finding, no apoptotic markers (cleaved forms of PARP and caspase-3) were detected in either of the BGJ398-treated GIST cells (Figure 4B). Similarly, FGFR inhibition also has no effect on the growth kinetics in IM-naive and -resistant GIST cells (Figure 4C). Strikingly, the FGFR inhibitor substantially enhanced the cytotoxic and pro-apoptotic effects of IM in GIST T-1R cells (Figure 4A,B, right panel). Moreover, in IM-resistant GIST cells, apoptotic markers were found only when both RTK inhibitors were used together. Similarly, IM supplemented with BGJ398 substantially affected the growth kinetics in IM-resistant GISTs, whereas each of RTK inhibitor used alone has no inhibitory effects on IM-resistant GIST cells (Figure 4C, left panel). In contrast, the FGFR inhibitor did not enhance IM-induced cytotoxicity and apoptosis in the IM-sensitive parental GIST T-1 cell line (Figure 4A,B, left panel).

Given that FGFR inhibition substantially reduced the viability of IM-resistant GIST T-1R cells when used in combination with IM (Figure 4, left panel) and taking into an account that multi-kinase inhibitor CB was effective in both IM-naive and -resistant GISTs (Figure 3), we examined whether the FGF-signaling pathway became attenuated in CB-treated GISTs. For this purpose, GIST T-1 vs. GIST T-1R cells were treated for 48 h with CB and BCG.

**Figure 5:**
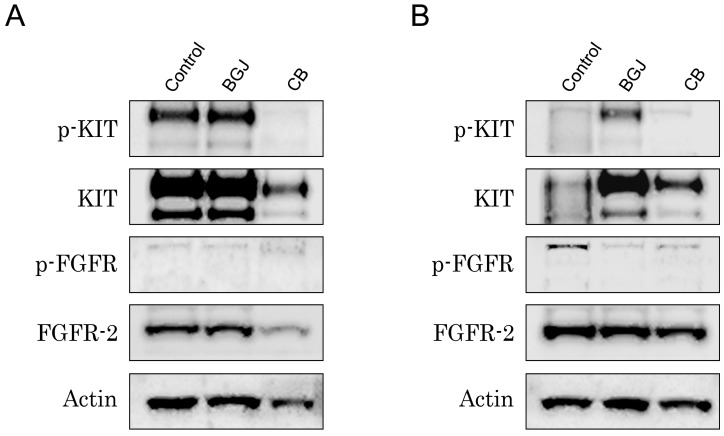
The effects of FGFR-inhibitor BGJ398 and cabozantinib (CB) on FGFR and KIT-signaling in IM-naive (**A**) and -resistant (**B**) GIST T-1 cells. Immunoblot analysis for total and phosphorylated forms of KIT and FGFR in non-treated (control), BGJ398- or CB-treated GIST T-1 and GIST T-1R cells. Actin stain was used as a loading control.

398 (as a positive control), and the expression of active, phosphorylated forms of KIT and FGFR, as well as total protein was examined by immunoblot. As expected, expression of phosphorylated and total KIT forms was substantially reduced in both GIST T-1 and T-1R cells after CB treatment (Figure 5A,B, respectively), whereas selective FGFR1-3 inhibitor has no similar effects on KIT activation. FGFR2 phosphorylation was detected in IM-resistant, but not in IM-naive GIST T-1 cells. As expected, BCG398 treatment effectively reduced the expression of phospho-FGFR2 in GIST T-1R cells (Figure 5B). Strikingly, CB treatment also provided a moderate inhibitory effect on FGFR2 phosphorylation in GIST T-1R cells, thus suggesting the novel molecular mechanism responsible for the CB inhibitory effect in IM-resistant GISTs with this type of RTK switch (loss of KIT/gain of FGFR).

Thus, we concluded that the inhibitory effects of CB in GIST T-1R cells were due to its dual molecular mode of action: inhibition of the FGF-signaling pathway and its well-known ability to inhibit c-MET, which became overexpressed in GIST T-1R cells (Figure 1B).

Of note, we also observed an increase of KIT phosphorylation in BGJ398-treated GIST T-1R cells, thus illustrating the cross-talk between KIT and FGFR signaling over the development of IM resistance in GISTs. Based on these data, we concluded that the type of RTK switch might be reversible. This point was also supported by our data illustrating that combined use of KIT and FGFR inhibitors was much more effective in IM-resistant GISTs when compared to the effects of CB and BGJ398 used alone (Figure 4, left panel).

Taken together, our data indicate that inhibition of the RTKs that became overexpressed in GISTs over the development of secondary IM resistance effectively reduces cellular viability of IM-resistant GISTs and, thus, might be considered as the prospective therapeutic targets for IM-resistant GISTs harboring this type of RTK switch (loss of c-KIT/gain of FGFR2α and/or c-MET).

### 2.4. IM-Resistant GISTs Are Less Sensitive to Certain DNA-Damaging Chemotherapeutic Agents

Since some of the DNA damaging agents were recently shown to be effective against GISTs cells in vitro and in vivo [22,23,24], we examined whether these drugs provide effective cytotoxic activities against the IM-resistant GIST T-1 subline lacking secondary KIT mutations and harboring the RTK switch. We found that the IM-resistant GIST subline was less sensitive to topoisomerase type II inhibitors, alkylating agents, tubulin-binding agents, etc. For example, in GIST T-1R cells, IC_50_ for paclitaxel increased 28-fold when compared to parental GIST T-1 cells (189.3 ± 17.3 and 6.7 ± 0.9, respectively). Similarly, a seven-fold difference in IC_50_ values between IM-resistant vs. sensitive GISTs was observed for etoposide (146.4 ± 7.0 and 20.7 ± 1.8, respectively). Given that decreased sensitivity of cancer cells to the multiple chemotherapeutic agents might be due to overexpression of the drug-efflux pumps that belong to the ATP-binding cassette (ABC) transporter superfamily, we examined the expression of the multidrug resistance (MDR)-associated transporter P-glycoprotein (MDR-1) or proteins from the MDR-associated protein (MRP) family in GIST T-1 vs. GIST T-1R cells. 

**Figure 6:**
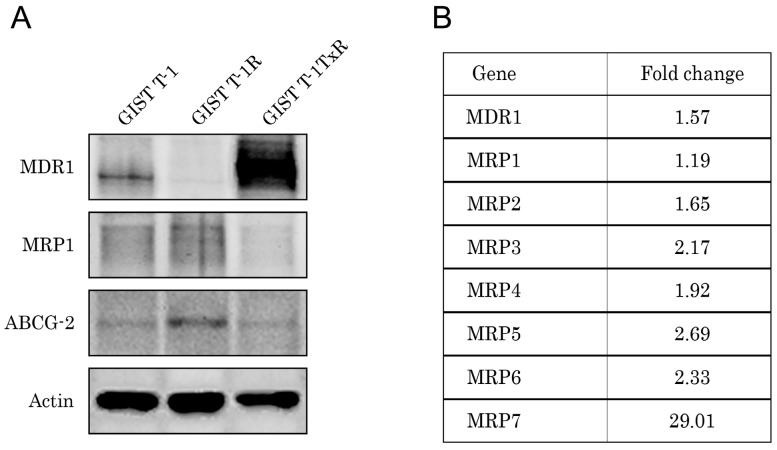
Multi-drug resistance (MDR) profile of GIST T-1R cells. (**A**) Expression of multi-drug resistance (MDR) proteins in GIST T-1 vs. GIST T-1R cells. Expression of P-glycoprotein (MDR-1) remained unchanged, whereas expression of ABCG2 and MRP-1 slightly increased in GIST T-1R cells when compared to parental GIST T-1 cells. The lysate of paclitaxel-resistant GIST T-1 cells (GIST-TxR) was used as a positive control for MDR-1 overexpression. Actin was used as a loading control; (**B**) Changes in the expression level of chemoresistance genes in GIST T-1R cells relative to GIST T-1 cells, as determined by reverse transcription quantitative polymerase chain reaction. GAPDH was amplified as an internal control.

Surprisingly, Western blot analysis showed no overexpression of the MDR-1 protein in GIST T-1R cells when compared with parental GIST T-1 cells (Figure 6A). As expected, the paclitaxel-resistant GIST T-1 cell subline, which has been previously established in our lab, exhibited a substantial increase of MDR-1 protein. Expression of the other proteins associated with the MDR phenotype, MRP-1 and ABCG2, showed only a minor increase in IM-resistant GIST cells when compared with IM-naive parental GIST T-1 cells. Similarly, no significant increase of P-glycoprotein mRNA and the majority of MRPs mRNAs (except MRP-7) was found in IM-resistant GIST T1-R cells when compared with parental GIST T-1 cells (Figure 6B). Consistent with these data, no significant differences in paclitaxel intracellular concentrations were found between IM-resistant GIST T1-R vs. GIST T-1 cells after 2 h of exposure to the chemotherapeutic agent (not shown). Taken together, these data indicate that the decreased sensitivity to the certain chemotherapeutic agents in GIST T-1R cells is not due to the increased activities of ABC-transporter-related proteins mediating an efflux of chemotherapeutic agents from GIST T1-R cells.

**Figure 7:**
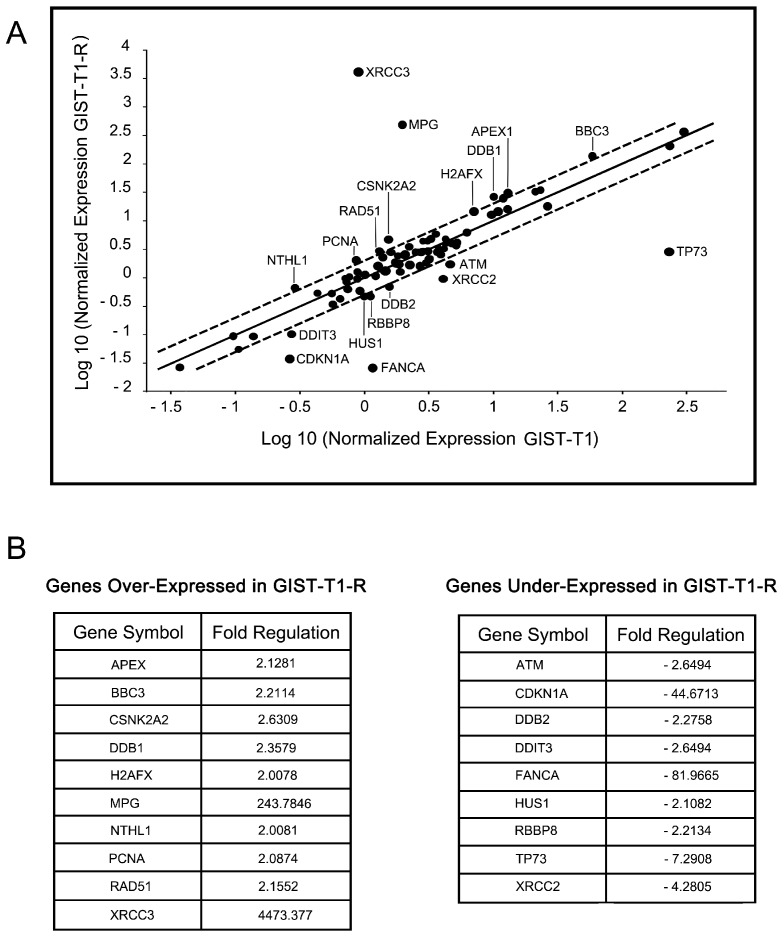
DNA damage repair (DDR) profile in GIST T-1R cells. (**A**) Relative expression comparison for 84 DDR-related genes between IM-resistant GIST T-1R (x-axis) and IM-sensitive parental GIST T-1 cells (y-axis). MPG, XRCC3 and CSNK2A2 genes were upregulated, while CDKN1A, FANCA, TP73 and XRCC2 were downregulated, by at least two-fold (outside the outer line) in GIST T-1R cells relative to the parental GIST T-1 cell line. Three genes (β2-microglobulin, β-actin and glyceraldehyde-3-phosphate dehydrogenase) were used for data normalization. (**B**) Changes in expression for DDR and cell cycle-related genes between IM-sensitive GIST T-1 and IM-resistant GIST T-1R cells. Genes from the experiment in Figure 6A that exhibited a two-fold or greater change in expression between GIST T-1 and GIST T-1R cells are presented.

To examine whether decreased sensitivity of IM-resistant GIST T-1 cells to the chemotherapeutic drugs is due to upregulation of DNA damage signaling and repair pathways (DDR), we conducted a global comparative analysis for genes involved in the various types of DNA damage signaling and repair (DDR) pathways, cell cycle regulation in both GIST T-1 and T-1R cells. For this purpose, we utilized a quantitative PCR Array Human DNA Damage Signaling Pathway (PAHS-029Z, SABiosciences, QIAGEN, Germantown, MD, USA). Of the 84 DNA damage signaling, repair and cell cycle arrest-focused genes in this array, 19 genes demonstrated at least an ~2-fold difference in gene expression between IM-sensitive parental GIST T-1 cells and the IM-resistant GIST T-1R derivate (Figure 7A). Upregulation was observed in 10 genes, while nine genes were downregulated in GIST T-1R cells (Figure 7B). For example, in the double-strand break (DSB) repair pathway, two genes were modestly (~2-fold) upregulated (Rad51, H2AFX), while four genes were downregulated (ATM, HUS1, RBBP8 and XRCC2) in GIST T1-R cells compared to the parental GIST T-1 cell line.

The most significant differences in DDR and signaling genes were found for MPG and XRCC3, which were substantially upregulated in GIST T-1R cells (243-and 4473-fold, respectively). It is well known that XRCC3 encodes a member of the RecA/Rad51-related protein family that participates in homologous recombination to maintain chromosome stability and repair DNA damage, thus suggesting that GIST T-1R cells might have a reduced sensitivity to topoisomerase type II inhibitors due to the substantial overexpression of XRCC3 protein and Rad51 recombinase, as well. At the same time, we observed a substantial (82-fold) decrease in the expression of FANCA in GIST T-1R cells when compared to the GIST T-1 cell line, thus indicating the potential sensitivity of IM-resistant GIST T-1R cells to DNA cross-linking agents due to the defective FANC-associated DNA repair pathway. We also detected a significant (44-fold) downregulation of CDKN1A expression in GIST T-1R cells when compared to parental GIST T-1 cells. CDKN1A (cyclin-dependent kinase inhibitor 1A) is known as a potent cyclin-dependent kinase inhibitor that mediates the p53-dependent cell cycle G1 phase arrest in response to a variety of DNA damaging stimuli. Of note, no significant changes in DDR and signaling genes were observed in GIST T-1R cells after BGJ398 treatment (data not shown), thus suggesting no potential relationship between changes in DDR signaling profile and activation of the FGF-signaling pathway in the IM-resistant GIST subline.

Taken together, we established and characterized the GIST T-R subline lacking secondary c-KIT and PDGFRA mutations typical for the IM-resistant phenotype and exhibiting resistance to IM due to the RTK switch (c-KIT/c-MET and FGFR2α). Indeed, MET and FGFR inhibitors were found more effective against IM-resistant GIST cells when compared to IM-sensitive GISTs. Importantly, these RTK inhibitors were able to enhance the cytotoxic effect of IM against IM-resistant GISTs. In addition, we also found that development of IM resistance in GIST cells might be associated with gene changes in DDR and the cell cycle regulation profile, thus making GIST cells less susceptible to certain chemotherapeutic agents. The resistance of GIST T-1R cells to certain chemotherapeutic drugs was not due to overexpression of ABC-related transporter proteins.

## 3. Discussion

It is well known that the common mechanism of GISTs’ resistance to IM includes second-site KIT mutations [25]. However, alternative molecular mechanisms involved in secondary IM resistance were recently described. These include hemi- or homo-zygous deletion of the wild-type Kit allele [6], overexpression of focal adhesion kinase (FAK) [7], insulin-like growth factor receptor I (IGF-1R) amplification [8], BRAF V600E mutation (5% GIST) [9], an RTK switch (loss of c-Kit and gain of MET/AXL) [10,15], activation of FGFR signaling, which attenuates the effects of IM in GISTs [11,12], etc. 

In this study, we established the IM-resistant GIST T-1 cell subline (GIST T1-R) from parental GIST T-1 cells. IM resistance was established over a 54-week period of GIST T-1 culturing in the presence of IM. The IC_50_ value for IM in GIST T1-R cells was >10,000-fold higher when compared with parental GIST T-1 cells (Figure 1A). IM resistance remained stable after one month of cell culture without targeted drug, or following storage at −80 °C for three months. 

As mentioned before, despite the initial clinical benefits of IM-based targeted therapy, most GIST patients develop IM resistance over two years due to secondary resistance mutations in KIT, thus suggesting that this time period is critical for the genetic changes responsible for secondary IM resistance in vivo. However, in vitro data illustrating the detailed molecular mechanisms of IM resistance in GISTs were missing. Tahakashi T. with colleagues recently published the intriguing data regarding the genomic and transcriptomic changes in several GIST T-1 sublines that acquired IM resistance in vitro [26]. Due to the exome sequencing analysis, the authors found that the GIST cell line that was briefly exposed to IM (for over seven weeks) exhibited only few single-nucleotide variants and copy-number alterations, but exhibited a significant upregulation of genes related to detoxification and downregulation of genes involved in cell cycle progression. In contrast, four resistant GIST cell lines that were exposed to IM for a longer time-period (up to 60 weeks) harbored numerous genomic changes: amplified genes related to detoxification and deleted genes with cyclin-dependent kinase activity. Strikingly, secondary KIT mutation (D820Y) was found only in two GIST cell sublines that were cultured in the presence of IM for 20 and 52 weeks, respectively, whereas the remaining 2 IM-resistant GIST sublines that were cultured with IM for 45 and 60 weeks did not harbor KIT mutations due to Sanger sequencing data. Of note, IC_50_ values for IM in all four GIST cell lines indicated above were >10 μM, thus indicating the diverse molecular mechanisms playing a role in IM resistance. Importantly, the duration of IM exposure is not a single factor involved in the secondary KIT mutations in GISTs.

Despite the long-time period (54 weeks) of GIST T-1 exposure to IM, the resistance to IM in our GIST T-1 subline was not due to the secondary KIT mutations. We found a substantial decrease of the basal expression of total and phosphorylated forms of KIT protein in GIST T-1R cells (Figure 1B), thus indicating that potential development of the RTK switch associated with the loss of KIT might be responsible for IM resistance in these cells. To examine this possibility, we tested whether aberrant hyperactivation of the other types of RTKs happened in GIST T-1R cells. For this purpose, we simultaneously assessed the activation status of the multiple receptor tyrosine kinases (RTKs) by using a low-scale phospho-proteome assay (Human Phospho-RTK Array Proteome Profiler™), which allows for the simultaneous detection of 42 RTKs in a single antibody-based microarray. Most strikingly, we observed a significant phosphorylation of FGFR2α in the IM-resistant GIST cells, whereas phosphorylation of KIT and PDGFRα was reduced when compared to IM-sensitive parental GIST cells, thus suggesting the novel type of RTK switch (loss of KIT and gain of FGFR) developed in IM-resistant GIST T1-R cells. There was no detectable phosphorylation of the remaining RTKs tested by this phospho-RTK array including ErbB1-3, FGFR1, FGFR3 and 4, IGF-1 receptor, Dtk, Mer, MSP receptor, PDGFRβ, Flt-3, M-CSF receptor, Axl, c-Ret, ROR1 and 2, Tie-1 and -2, TrkA, B and C, VEGFR1–3, MuSK, EphA1–7 and EphB1, 2, 4 and 6.

Thus, we found a phospho-FGFR2α that became significantly upregulated in IM-refractory GIST cells. To our knowledge, this is a novel finding suggesting the prospective use of FGFR inhibitors for IM-resistant GISTs lacking second-site KIT mutations and having this specific type of RTK switch (loss of c-KIT/gain of FGFR2α). Of note, silencing of fibroblast growth factor receptor 3 (FGFR3) was recently found to have substantial inhibitory effects on proliferation and cell viability of IM-resistant GIST cells [10]. Moreover, the experiments utilized the inhibition and silencing of FGFR3, and KIT highlighted the existence of the cross-talk between these RTKs in IM-resistant GIST cells [11].

Despite no significant difference between GIST T-1 and T-1R cells being found in phospho-MET expression, our Western blot analysis indicated a substantial increase of the total form of MET in IM-resistant GIST cells. Importantly, after IM exposure, we observed the decreased expression of total MET, whereas the expression of the phosphorylated form of this RTK became increased in both GIST cell lines (Figure 1B). This is consistent with the previous report indicating the ability of IM to activate MET-signaling in GIST cells [16].

To examine the prospective use of RTK inhibitors to treat the subset of patients with GISTs lacking secondary KIT/PDFRA mutations and having an RTK switch (loss of c-KIT/gain of MET and/or FGFR2α), the following RTK inhibitors were tested in the present study: (a) crizotinib (CR), c-MET/ALK inhibitor; (c) cabozantinib (CB), a multi-kinase c-KIT/c-MET/VEGFR2,3 inhibitor; (c) BGJ398, a selective FGFR inhibitor for FGFR1-3.

We observed a dose- and time-dependent inhibition of viability and proliferation in IM-sensitive and -resistant GIST T-1 cells after CB exposure. Importantly, selective MET and FGFR inhibitors (CR and BGJ398, respectively) used alone have minor or no inhibitory effects on viability and proliferation in IM-sensitive GIST T-1 cells, thus supporting the lack of activation of these RTKs (MET and FGFR) in IM-naive GIST cells (Figure 2A, Figure 3A and Figure 4A). Importantly, CR when used alone inhibited proliferation and viability in GIST T-1R cells. In contrast, FGFR inhibition has no similar effects in GIST T-1R cells. Strikingly, when each of the RTK inhibitors indicated above was used in combination with IM, a decrease of proliferation and viability was observed. These data are consistent with the recent findings illustrating that the combination of KIT and FGFR inhibition increased the growth inhibition in IM-sensitive GIST cell lines (e.g., GIST-T1 and GIST882) and improved efficacy in patient-derived GIST xenografts [11]. We also found that selective FGFR1-3 inhibitor BGJ398 has no effect on cell viability and growth of IM-sensitive GIST T-1 cells, which was consistent with the previous report [11]. However, we observed that FGFR inhibition moderately reduced the viability of IM-resistant GIST T-1R cells, thus indicating that the activation of the FGF-signaling pathway in IM-resistant GISTs acquired this type of RTK switch. Strikingly, dual inhibition of FGFR and KIT-signaling in IM-resistant GISTs has a substantial effect on GISTs’ viability and proliferation and effectively induced apoptosis. (Figure 4, left panel).

Importantly, we also observed the decrease of FGFR2 phosphorylation in CB-treated GIST T-1R cells, thus suggesting the novel molecular mechanism responsible for CB’s effectiveness in IM-resistant GISTs exhibiting this specific type of RTK switch (loss of KIT/gain of FGFR). Given that the impact of selective c-MET inhibitor CR on GISTs’ viability was modest, even when combined with IM and taking into the account that CB was much more potent when compared to CR, we concluded that activation of FGFR, but not the MET-signaling, pathway was the major driving component of IM resistance in GIST T-1R cells. 

Of note, we found that IM-resistant GIST T-1 cells are less sensitive to certain DNA-damaging chemotherapeutic agents, e.g., topoisomerase type II inhibitors, alkylating agents and tubulin-binding agents. The resistance was not due to overexpression of the multidrug resistance (MDR)-associated transporter P-glycoprotein (MDR-1) or proteins from the MDR-associated protein (MRP) family. Indeed, we observed a similar intracellular concentration of chemotherapeutic agents (e.g., paclitaxel) in IM-resistant GIST T1-R vs. GIST T-1 cells after 2 h of exposure to the chemotherapeutic agents, thus indicating no increase of drug efflux from IM-resistant GIST cells.

FGF2 (over)expression in tumor and non-tumor cells was observed after chemo- and radio-therapy, associated with radio- and chemo-resistance, correlated with an increased risk of recurrence and reduced overall survival [27,28,29,30]. Thus, the resistance of GIST T-1R cells to certain chemotherapeutic agents might reflect the activation of FGF2-mediated autocrine mitogenic and pro-survival signals, mediating the resistance to the chemotherapeutic drugs.

We found that several genes involved in multiple DNA damage signaling and repair pathways became upregulated in GIST T-1R cells when compared to the parental GIST T-1 cells. The most substantial increase (~4500-fold) was found for XRCC3, which encodes a member of the RecA/Rad51-related protein, a paralog of the strand-exchange protein RAD51 [31] involved in homology-mediated repair (HR) of double-strand breaks (DSBs) in mammalian cells [32]. A moderate (~2-fold) increase of the other genes encoding the HR-related proteins, such as Rad51, H2AFX, etc., was also found in IM-resistant GIST cells. We also detected a significant (~250-fold) upregulation of MPG, which encodes a 3-alkyladenine DNA glycosylase (AAG) or *N*-methylpurine DNA glycosylase (MPG), an enzyme involved in the recognition of a broad spectrum of base lesions, including alkylated and deaminated purines, and initiating the repair via the base excision repair (BER) pathway. Taken together, these data suggest that reduced sensitivity to the alkylating agents, topoisomerase type II inhibitors and other certain chemotherapeutic drugs in IM-resistant GIST cells might be due to the hyperactivation of the DNA damage signaling and repair pathway(s).

In conclusion, we established the GIST T-1R subline, which is highly resistant to IM and harbors a cross-resistance to the diverse types of chemotherapeutic agents with similar and different modes of action, as well. Importantly, GIST T-1R cells lack secondary KIT mutations and overexpress c-MET and phospho-FGFR2α, thus being sensitive to the corresponding RTK inhibitors used alone or in combination with IM. Taken together, the established GIST T-1R cell line might be effectively used for in vitro and in vivo studies to examine the selective MET and FGFR inhibitors as the prospective agents to improve the treatment and prognosis of GIST patients who acquired IM resistance due to this type of RTK switch.

## 4. Materials and Methods

### 4.1. Chemical Compounds 

Imatinib (IM), cabozantinib (CB), crizotinib (CR) and BGJ 398 were obtained from SelleckChem (Houston, TX, USA). Doxorubicin (Dox), paclitaxel (PTX) and vinblastine (Vin) were purchased from Sigma (St. Louis, MO, USA). Etoposide (Eto) was obtained from Calbiochem (La Jolla, CA, USA). 

### 4.2. Antibodies

Primary antibodies used for immunoblotting were as follows: total and cleaved forms of PARP and caspase-3, phospho-KIT Y719, phospho-AKT S473, AKT, ABCG2, phospho-FGFR Y653/654 (Cell Signaling, Danvers, MA, USA), FAK, phospho-FAK Y397 (BD Biosciences, Franklin Lakes, NJ, USA), KIT (DakoCytomation), phospho-Met Tyr1230/1234/1235 (R&D Systems, Minneapolis, MN, USA), Axl, FGFR2 (Sigma), c-Met, MDR, MRP-1 and actin (Santa Cruz Biotechnology, Dallas, TX, USA). The HRP-conjugated secondary antibodies for Western blotting were purchased from Santa Cruz Biotechnology.

### 4.3. Cell Lines and Culture Conditions

The human IM-sensitive GIST T-1 cell line was used in the present study. GIST T-1 was established from a metastatic plural tumor from the stomach GIST and contains heterozygous 57-base pair deletion (V570-Y578) in KIT exon 11 [33]. GIST T-1 cells were maintained in RPMI-1640 medium supplemented with 10% and 15% fetal bovine serum (FBS), respectively; 1% l-glutamine, 50 U/mL penicillin, 50 µg/mL streptomycin. The IM-resistant GIST T-1R subline was established in our laboratory after a continuous induction from 0.4 nM–1000 nM IM in a stepwise increasing concentration manner (see below). The cell lines indicated above were cultured in a humidified atmosphere of 5% CO_2_ at 37 °C (LamSystems, Miass, Russia).

### 4.4. Cellular Survival MTS-Based Assay

GIST cells indicated above were seeded in 96-well flat-bottomed plates (Corning Inc., Corning, NY, USA) and allowed to attach and grow for 24 h. The cells were then cultured for 24–48 h with indicated concentrations of the RTK inhibitors or chemotherapeutic agents or dimethyl sulfoxide DMSO only (control).

Finally, 3-(4,5-dimethylthiazol-2-yl)-5-(3-carboxymethoxyphenyl)-2-(4-sulphophenyl)-2*H*-tetrazolium, inner salt (MTS) reagent (Promega, Madison, WI, USA) was added to the culture medium to assess the live cell numbers. The cells were incubated with MTS reagent for at least 1 h and assayed at 492 nm on a MultiScan FC plate reader (Thermo Fisher Scientific, Waltham, MA, USA). Resulting IC_50_ values were defined as the compound concentration required to inhibit cellular growth by 50% in 24–48 h. The data were normalized to the DMSO-treated control group.

### 4.5. Real-Time Monitoring of Cell Proliferation 

The growth curves of GIST cells cultured in the presence of RTK inhibitors and/or chemotherapeutic agents were analyzed by using iCELLigence system (ACEA Biosciences, San Diego, CA, USA). For this, GIST cells were seeded in electronic microtiter plates (E-Plate; Roche Diagnostics, GmbH, Mannheim, Germany) for 24 h to obtain the growth baseline reading. Next, the cells were pre-treated with IM, sunitinib (SU) or DMSO (control) for 12 h in triplicates and further treated with Dox, Eto or DMSO (control) for 48–72 h. Cell index (CI) measurements were performed with a signal detection set for every 30 min until the end of experiment (72 h). Normalized cell index (NCI) values were analyzed by RTCA software (Roche Diagnostics, GmbH, Mannheim, Germany).

### 4.6. Western Blotting Analysis 

For Western blotting analysis, whole-cell extracts were prepared by scrapping the cells growing as a monolayer into RIPA buffer (1% NP-40, 50 mM Tris-HCl pH 8.0, supplemented with protease and phosphatase inhibitors). The cellular lysates were incubated for 1 h at 4 °C and then clarified by centrifugation for 30 min at 13,000 rpm at 4 °C. Protein concentrations were measured by the Bradford assay. The samples containing 30 μg of protein were resolved on 4–12% Bis-Tris or 3–8% Tris-acetate NuPAGE gels (Invitrogen, Carlsbad, CA, USA), transferred to a nitrocellulose membrane (Bio-Rad, Hercules, CA, USA), probed with specific antibodies and visualized by enhanced chemiluminescence (Western Lightning Plus-ECL reagent, Perkin Elmer, Waltham, MA, USA). Densitometric analysis of Western blotting images was performed by using the NIH ImageJ software (Bethesda, MD, USA), which was downloaded from the NIH website (http://rsbweb.nih.gov/ij/download.html).

### 4.7. Phosphokinase Arrays

The Proteome Profiler Human Phospho-Kinase Array Kit (R&D Systems, Inc., Minneapolis, MN, USA) was used to measure protein phosphorylation in GIST cell lines. Briefly, 50 μg of protein were applied for the array and captured by antibodies spotted on a nitrocellulose membrane. Levels of the phospho-protein expression were then examined by using an HRP-conjugated secondary antibody followed by chemiluminescence detection. The levels of chemiluminescence were detected and analyzed using the corresponding array software. 

### 4.8. DNA Damage Signaling RT Profiler PCR Array 

Total RNA was isolated either from GIST T-1 and GIST T-1R cells using the TRIzol reagent (RNAiso, Invitrogen, Carlsbad, CA, USA). RNA was extracted with phenol-chloroform, ethanol precipitated and resuspended in DEPC-treated water. Total RNA from GIST T-1 and T1-R cells was converted into cDNA using the RT2 First Strand Kit (SABiosciences, Frederick, MD, USA). This cDNA was then added to the RT2 SYBR Green qPCR Mastermix (SABiosciences, Frederick, MD, USA). Next, each sample was aliquoted in equal volumes to each well of the real-time PCR arrays (SABiosciences, PAHS-029Z, Frederick, MD, USA) and harvested per the manufacture’s protocol. The Human DNA Damage Signaling Pathway RT2 Profiler PCR Array (SABiosciences, Frederick, MD, USA) interrogates 84 genes related to the DNA damage response. The real-time PCR cycling program was run on a CFX 96 Real-Time detection system (Bio-Rad, Hercules, CA, USA). The threshold cycle (Ct) of each gene was determined and subsequently analyzed by RT2 Profiler PCR Array data analysis software downloaded from the manufacturer’s website (http://www.sabiosciences.com/pcrarraydataanalysis.php).

### 4.9. Genetic Analysis

Total DNA was extracted by using the NucleoSpin Tissue kit (Macherey Nagel). DNA concentration was determined using a Qubit double-stranded DNA High Sensitivity Assay Kit and Qubit fluorometer (ThermoFisher Scientific, Waltman, MA, USA). Secondary mutations in the KIT gene were determined using the TruSight™ Cancer Sequencing Panel (Illumina, San Diego, CA, USA) according to the manufacturer’s instructions. Captured libraries were assessed for quality using the Agilent High Sensitivity DNA assay and yield using the Qubit double-stranded DNA High Sensitivity Assay Kit and the Qubit fluorometer (ThermoFisher Scientific, Whatman, MA, USA). The resulting library was sequenced using the Illumina MiSeq platform and MiSeq Reagent Kit v 2 (300 cycles) to generate 149-bp paired-end reads (2 × 149 PE). (“Illumina”) following the manufacturer’s protocol. Raw-data reads were aligned to the human reference genome (hg 38) using aligner BWA-MEM with BamQC, FastQC and NGSrich quality control checks. Single nucleotide polymorphism (SNP) analysis was performed using CLC Medical Workbench 3.0.

### 4.10. Statistics 

All experiments were repeated 3 times. The results are presented as the mean ± standard error (SE) for each group. The Kaplan–Meier test was used for survival analysis. Differences were considered significant at *p* < 0.05.
